# Decision regret analysis in early URSL vs medical expulsive therapy 1 for ureteric calculi ≤ 1cm

**DOI:** 10.1007/s00345-024-05228-2

**Published:** 2024-10-03

**Authors:** Anshuman Singh, Arun Chawla, Kasi Viswanath Gali, Sunil Pillai Bhaskara, Padmaraj Hegde, Charan Kothuri, Jean de la Rosette, Pilar Laguna

**Affiliations:** 1 Department of Urology and Renal Transplant, Kasturba Medical College, Manipal, Manipal Academy of Higher Education, Manipal, India; 2 Department of Urology, Istanbul Medipol University, İstanbul, Turkey

**Keywords:** Shared decision making, Ureteroscopy, Ureteroscopic lithotripsy, URSL, Medical expulsive therapy, MET, Quality of life, QoL

## Abstract

**Background:**

The study assesses the decisional regret following Shared Decision-making (SDM) in patients selecting either early ureteroscopic lithotripsy (URSL) or medical expulsive therapy (MET) for ureteric stones ≤ 1 cm, with the aim to evaluate their decisional Conflict, satisfaction, and regret regarding their opted treatment choices.

**Methods:**

Adults aged more than 18 years with one stone up to 1 cm in either ureter were included. After SDM, the patients were allocated into their opted group viz. URSL or MET. Patients in each group were reassessed at “treatment completion”. Cambridge Ureteric Stone PROM (CUSP) questionnaire for HRQoL, Decision Regret Scale and the OPTION scale (SDM) were filled at treatment completion.

**Findings:**

111 patients opted for MET, while 396 patients opted for early URSL. Mean stone size was larger in URSL group (7.16 ± 1.63 mm vs. 5.50 ± 1.89; *p* < 0.001). Decisional conflict was higher in patients opting for URSL (77.3% vs. 57.7%; *p* < 0.001). Stone-free rate at four weeks was higher in URSL group (87.1%vs68.5%, *p* < 0.001). Decisional regret was higher in patients opting for MET (33.24 ± 30.89 vs. 17.26 ± 12.92; *p* = 0.002). Anxiety, was higher in patients opting for MET (6.94 ± 1.89 vs. 5.85 ± 1.54; *p* < 0.001). Urinary symptoms and interference in patients’ travel plans and work-related activities were more in URSL group (6.21 ± 1.57 vs. 5.59 ± 1.46; *p* < 0.001 and 6.56 ± 1.59 vs. 6.05 ± 1.72; *p* < 0.001 respectively).

**Interpretation:**

After SDM, decisional regret is higher in patients opting for MET mainly due protracted treatment duration with increased pain and anxiety during the treatment course and the need for additional procedure for attaining stone clearance and the. Despite higher decisional conflict, a larger proportion of patients opt for early URSL with the aim of avoiding anxiety and achieving early stone clearance.

**Supplementary Information:**

The online version contains supplementary material available at 10.1007/s00345-024-05228-2.

## Introduction

Patients presenting with ureteric colic must choose between ureteroscopic lithotripsy (URSL), extracorporeal shockwave lithotripsy (ESWL), medical expulsive therapy (MET) or observation [1, 2]. Decision-making between these options traditionally relies on the likelihood of achieving stone-free status and the potential for complications [3–5]. There is an evolving emphasis on patient experiences in urolithiasis on treatment decisions [6–14]. Guidelines strongly recommend the use of shared decision making (SDM) for selection of treatment modalities for ureteric calculi [15]. Patient experiences and Quality of life may significantly differ for each option, highlighting the need for evaluating patient perspectives as a crucial aspect of treatment selection.

Our study compares decisional conflict, quality of life outcomes, and decisional regret in patients choosing between URSL and MET after shared decision making for the management of ureteric calculi. The primary objective is to compare decisional regret between the groups. Secondary objectives include comparing health-related quality of life (HRQoL), decisional conflict, stone-free rates at four weeks, and the need for additional procedures for stone clearance.

## Methods

### Study design and participants

We conducted a prospective cohort study from June 2021 to January 2023> in which 721 patients with suspected ureteric colic were evaluated and 651 were confirmed to have ureteric calculus via CT KUB. Adult patients aged ≥ 18 years presenting with uncomplicated ureteric calculi ≤ 1 cm in the largest dimension were included in the study. Patients needing immediate intervention due to secondary complications of calculus including sepsis and acute kidney injury patients with abnormal renal anatomy bilateral ureteric calculi calculus in the ureter of solitary functional kidney estimated glomerular filtration rate of less than 60 ml/min/1.73m^2^, those already taking or unable to take an α-blocker (planned to be given as a part of medical expulsive therapy) pregnant females asymptomatic incidentally detected ureteric calculus more than one calculus in a single ureter and patients with known allergy to tamsulosin diclofenac or drotaverine were excluded from the study. Out of 651 patients confirmed to have ureteric calculus 144 were excluded for not meeting the inclusion criteria. For the remaining 507 patients engaged in SDM 111 opted for medical expulsive therapy (MET) while 396 chose early URSL. The response rates for initial and follow-up questionnaires were 85% and 71% respectively with missing data imputed by the hot deck method.

### Procedures

Patients who fulfilled the inclusion criteria were explained about the process of SDM and the available modalities for the treatment of ureteric calculus. Treatment options offered to the patients were early URSL, trial of MET and ESWL. Safety, efficacy and complication rates for each option was explained as per the most recent evidence available in literature. Patients opting for ESWL as the primary treatment modality were excluded from the study. Those patients who opted for either early URSL or trial of MET were assessed for the presence of decisional conflict for making the informed healthcare related decision after SDMusing theSURE(Sure of myself Understand information Risk-benefit ratio Encouragement) score given in the form of a 4 item self-administered questionnaire.

Patients who opted for early ureteroscopy were taken up for procedure within twenty four hours of hospital admission while those who opted for a trial of MET were discharged with advice for self-administered tamsulosin 0.4 mg to be taken daily for four weeks along with combination of aceclofenac 100 mg and drotaverine 80 mg (administered as separate oral tablets) to be taken during episode of ureteric colic. Patients in each group were reassessed at “treatment completion.” Treatment completion in MET group was defined as absence of pain at the end of four weeks along with no evidence of stone on combined abdominal Ultrasonography and X-ray KUB. Treatment completion in early URSL group was defined as the point when the patient becomes “stone free” and “stent free”. Stone free status was defined by absence of stone or its fragment during endoscopic and fluoroscopic assessment during procedure and on follow-up with combined imaging by ultrasonography with X-ray KUB done at the time of stent removal. Stone fragments of size 2 mm or less, as assessed by its visual comparison with the lithotripsy probe / Laser fiber, if migrated to kidney at the time of early URSL, were considered clinically insignificant and no additional procedure was performed for attempting clearance.

### Outcomes

The primary outcome was decisional regret for the opted modality after SDM. The secondary outcomes were decisional conflict at the time of SDM, HRQoL during the treatment duration and the need for additional procedures for treatment completion.

**Fig. 1:**
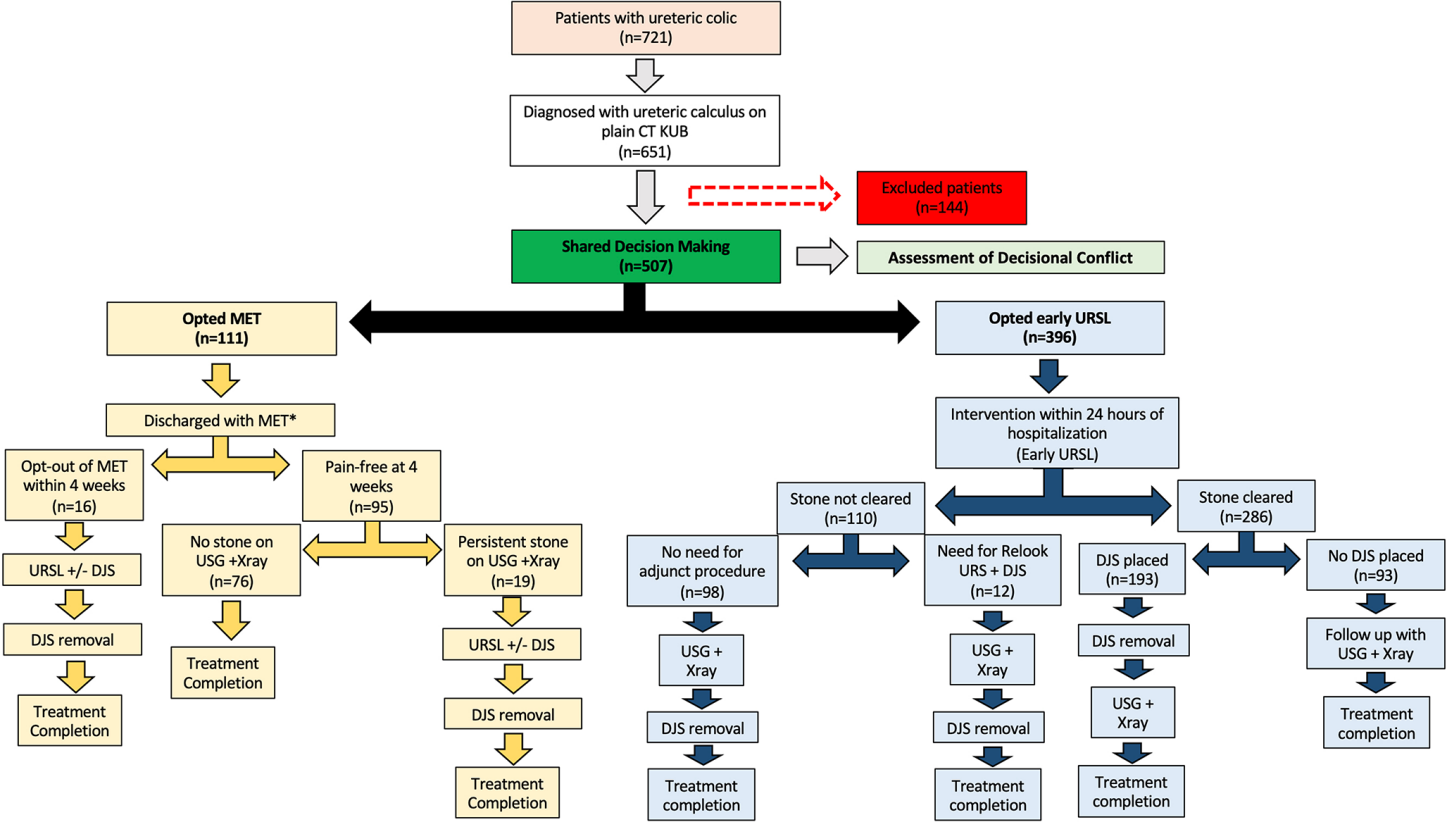
Flow diagram of the study

At treatment completion the patients were asked to fill the CUSP (Cambridge Ureteric Stone Patient Reported Outcome Measure) questionnaire to assess the impact of entire treatment course on the patients HRQoL, which is a specific measure of the impact of the health related condition on the patients overall quality of life. Decision Regret Scale (DRS) questionnaire was used to assess for patients’ satisfaction and regret after taking the healthcare related decision after shared decision making. OPTION (Observing Patient Involvement) scale which assesses the extent of SDM between healthcare providers and patients was used for assessing the patients’ opinion regarding the extent to which they felt involved in taking the decision thereby reflecting on the quality of shared decision making with respect to each treatment modality. To ensure maximal response rate, the questionnaires were administered in the form of physical sheets, online form embedded into a link delivered through text message or email. The missing data for the questionnaire responses was imputed using hot deck method. The study methodology has been represented in Figure 1.

### Statistical methods

In data analysis, continuous variables were summarized with means and standard deviations or medians and interquartile ranges, while categorical variables were described using frequencies and percentages. Data visualizations were employed selectively for enhanced clarity. Independent sample ‘t-tests’ and non-parametric Wilcoxon Tests were applied for two-group comparisons of continuous data. Categorical data comparisons relied on Chi-squared or Fisher’s Exact tests, depending on expected frequencies in contingency tables. Correlations between continuous variables were assessed using Pearson’s or Spearman’s tests, based on data distribution. Paired t-tests and Wilcoxon Signed Rank tests were used for paired analyses. For multiple group comparisons of continuous data, ANOVA or Friedman tests were utilized. A p-value of less than 0.05 was considered statistically significant.

### Ethical considerations and funding

#### Ethical approval

for this study was granted by the institutional ethics committee (EC/NEW/INST/2021/1707), adhering to the principles of the Declaration of Helsinki, and it was registered with the Clinical Trials Registry - India (CTRI/2022/03/041488). All participants provided written informed consent in accordance with institutional guidelines. The study was conducted without any funding received by the authors.

## Results

The URSL group had a larger mean stone size (7.16 ± 1.63 mm) than the MET group (5.50 ± 1.89 mm, *p* < 0.001). A greater proportion of the URSL group had stones over 5 mm (77.8%) compared to the MET group (50.5%, *p* < 0.001), with most stones located in the distal ureter without significant distribution difference between groups. Stone clearance on the first procedure was 72.6% for URSL, with 77.1% receiving a double J stent. In the MET group, 14.9% discontinued due to pain. After four weeks, stone clearance was higher in the URSL group (87.1%) than in the MET group (68.5%, *p* < 0.001), with the latter requiring more additional procedures (31.5% vs. 3.0% in the URSL group, *p* < 0.001). (Table I)

The decisional conflict score was higher in the URSL group (77.3%) compared to the MET group (57.7%, *p* < 0.001). In terms of quality of life (QoL), the URSL group reported less pain (mean score 16.17 ± 4.02) than the MET group (16.51 ± 4.20, *p* = 0.026), more anxiety in the MET group (6.94 ± 1.89 vs. 5.85 ± 1.54 in the URSL group, *p* < 0.001), and higher disruption to work and daily activities in the URSL group (6.56 ± 1.59 vs. 6.05 ± 1.72 in the MET group, *p* = 0.0011). Urinary symptoms were also more significant in the URSL group (6.21 ± 1.56 vs. 5.59 ± 1.46 in the MET group, *p* < 0.001). The MET group exhibited higher decision regret (33.24 ± 30.89) compared to the URSL group (17.26 ± 12.92, *p* = 0.002), with no significant difference in the OPTION score for patient involvement in decision-making (*p* = 0.9). (Table I)

Propensity score matched analysis (111 patients per group i.e. URSL & MET).

The URSL group reported higher decisional conflict (*p* < 0.001) but achieved a significantly better stone clearance rate at 4 weeks (*p* < 0.001), required fewer additional procedures (*p* < 0.001), lower decisional regret (*p* = 0.004) and lower anxiety (*p* < 0.001). (Supplementary Table II)

A Subgroup analysis was performed with patients having stones ≤ 5 mm and > 5 mm. URSL patients with stones ≤ 5 mm had significantly lower decisional regret (*p* = 0.001). (Supplementary Table III).

### Post hoc statistical power calculation

For post hoc power calculation for the whole sample, a one-tailed t-test was run to ascertain the presence of a significant difference between two groups at a chosen level for statistical significance set at 0.05 (α error probability). The observed effect size (d) was found to be approximately 0.78. The non-centrality parameter (δ) was calculated to be 7.19. The critical t-value at the 0.05 significance level, with 478 degrees of freedom (df), was determined to be 1.65. The calculated statistical power was high at 0.998, which equates to 99.8%.

**Table 1:** Comparative analysis of the two groups

Parameters	Group		*p* value
	URSL	MET	
**Number of patients (n)**	396	111	
**Age (Years)**	38.61 ± 13.54	39.69 ± 14.03	0.492^1^
**Gender**			0.983^2^
Male	325 (82.1%)	91 (82.0%)	
Female	71 (17.9%)	20 (18.0%)	
**Side Of Calculus**			0.354^2^
Right	230 (58.1%)	59 (53.2%)	
Left	166 (41.9%)	52 (46.8%)	
Size Of Calculus (mm)*	7.16 ± 1.63	5.50 ± 1.89	< 0.001^1^
Size Of Calculus*			< 0.001^2^
≤ 5 mm	88 (22.2%)	55 (49.5%)	
> 5 mm	308 (77.8%)	56 (50.5%)	
**Location**			0.233^2^
Proximal	65 (16.4%)	25 (22.5%)	
Mid	84 (21.2%)	18 (16.2%)	
Distal	247 (62.4%)	68 (61.3%)	
**Stone Cleared at First Procedure (Yes)**	286 (72.8%)	-	-
**Stent Deployed (Yes)**	303 (77.1%)	-	-
**Opt-Out of MTE (Yes)**	-	16 (14.4%)	-
Stone Clearance at 4 Weeks (Yes)*	343 (87.1%)	76 (68.5%)	< 0.001^2^
Need For Additional Procedure (Yes)*	12 (3.0%)	35 (31.5%)	< 0.001^2^
Decisional Conflict (Yes)*	306 (77.3%)	64 (57.7%)	< 0.001^2^
**CUSP Questionnaire**			
Pain*	16.17 ± 4.02	16.51 ± 4.20	0.026^1^
*Fatigue*	10.37 ± 2.47	10.44 ± 2.97	0.862^1^
*Alteration in Work*, Daily Activities & Travel Plans*	6.56 ± 1.59	6.05 ± 1.72	< 0.001^1^
*Sleep Disturbance*	7.98 ± 1.93	7.78 ± 2.15	0.256^1^
Anxiety*	5.85 ± 1.54	6.94 ± 1.89	< 0.001^1^
Urinary Symptoms*	6.21 ± 1.57	5.59 ± 1.46	< 0.001^1^
Decision Regret Score*	17.26 ± 12.92	33.24 ± 30.89	0.002^1^
**OPTION Tool score**	71.91 ± 9.56	71.99 ± 9.64	0.937^1^

Table legends: URSL: Ureteroscopy Lithotripsy; MET: Medical Expulsive Therapy; OQ: OPTION Questionnaire; HRQoL: Health-Related Quality of Life; mm: Millimeters; NA: Not Applicable

1: Wilcoxon-Mann-Whitney U Test, 2: Chi-Squared Test

* Statistically significant at p < 0.05

## Discussion

Significantly higher proportion of patients opted for early ureteroscopy as the treatment of choice over a trial of MET. Early URSL has better results in terms of patient centred outcomes. It is associated with better satisfaction rate due to early definitive treatment of stone, high stone clearance rates and less anxiety related to fear of recurrent colic episodes and stone related complications.

After SDM, URSL was the preferred treatment over MET, reflecting literature that links treatment choices to more successful outcome [16]. The invasiveness of URSL did cause more decisional conflict, yet its higher selection rate suggests a patient bias towards avoiding the recurrence of pain and frequent emergency hospital visits over the risks of an invasive procedure. The correlation between larger stone sizes and the choice of URSL likely points to the communicated lower efficacy of MET for larger stones during SDM sessions.

Decisional conflict denotes the uncertainty patients feel when making tough healthcare choices. In our study, those opting for URSL faced greater difficulty deciding, possibly due to the procedure’s invasiveness, associated risks, anaesthesia concerns, and recovery duration. The precise causes of this increased decisional conflict remain unexplored, highlighting a need for further research into these specific factors (Table 1).

In our study, we mirrored a real-world clinical scenario by allowing patients with ureteric colic to choose between early URSL and MET, facilitating a more natural SDM process. We determined treatment success by the absence of further treatment interventions, thereby minimizing unnecessary radiation from repeat CT scan. Using this strategy, the likelihood of overlooking asymptomatic residual stones was considered to be minimal [17]. In the URSL group, fragments of size 2 mm or less were considered clinically insignificant as they pose minimal risk of recurrent colic or complications [18]. The study’s pragmatic design enhances the applicability of the results by comparing the two treatment modalities in practical scenarios, unlike RCTs that operate under more controlled but less real-world conditions.

CUSP-Questionnaire responses indicated that MET patients experienced higher pain and anxiety, mainly due to uncertainty over outcome, implying a potentially larger psychological impact compared to those who chose early URSL. Conversely, URSL patients reported more disruption to work and daily life, underscoring the importance of considering lifestyle impacts in treatment decisions (Table I). The increased disruption to work and daily life in the URSL group could be attributed to the self-imposed restriction and leave from work in URSL patients due to the influence of the operative procedure, despite early discharge and encouragement for early return to normal activities following the procedure.

Subgroup analysis of stones ≤ 5 mm revealed that URSL patients had significantly lower decisional regret, suggesting greater satisfaction with this choice. For stones > 5 mm, the MET group’s regret scores were notably higher, indicating more dissatisfaction likely due to the non-invasive approach’s failure to quickly resolve symptoms, exacerbating regret when additional interventions became necessary. These comprehensions suggest that patients who are particularly anxious or concerned about the potential need for additional treatments, risk of recurrent colic or the protracted duration of treatment required for achieving stone clearance, may be better served by early URSL. Clinicians should recognize the potential for decisional conflict and regret and address them using decision aids and comprehensive shared decision-making strategies [19].

This study has several strengths, including its large sample size and the use of validated measures to assess decisional conflict and patient-reported outcomes. When multiple treatment modalities with equally or nearly equivalent outcomes are available, patient perspectives play a major role in making an informed decision for the selection of treatment. While the impact of treatment choice on patient outcomes, such as treatment regret and health-related quality of life, is well established for prostate cancer [20, 21], its effects on patients with ureteric stones are less understood. A patient who comprehends the logic of treatment and follow-up is more prepared to translate treatment plans into a workable daily routine of disease management [22]. With the development of a variety of more efficacious treatment approaches, the clinical emphasis on assessing patient’s preferences and discussing different treatment options has become increasingly important [22]. The benefits of increased patient participation in treatment plans have already been demonstrated in literature [23, 24]. Previous studies have reported patient outcomes with self-selected therapy for ureteral stones, but have not evaluated why patients make decisions with regards to which treatment approach is best for them [25]. This study provides an innovative insight into patient-centric outcomes during ureteric colic treatment in real-world settings. Its findings have the potential to improve patient decision aids for ureteric stone treatment by offering crucial patient-centric data, thereby assisting future patients in making well-informed decisions.

Our study’s limitations include its reliance on questionnaires and the lack of data on employment, insurance, and income, which could influence patient decision-making. The sole recruitment from a University Teaching Hospital may not reflect the broader population, particularly first-time stone formers, due to participants’ potential prior stone experiences and interventions. Additionally, follow-up evaluations of MET patients used USG or X-ray instead of CT, reducing radiation exposure but possibly overlooking some asymptomatic retained stones. Exclusion of patients opting for ESWL in an attempt to provide direct comparison between two treatment modalities can also be considered as another limitation of the study.

## Conclusion

After SDM for the treatment of an uncomplicated small ureteric calculus, early ureteroscopic intervention is the preferred modality for patients as compared to a trial of MET. This preference could be attributed to early definitive treatment of stone and reduced risk of recurrent colic episodes. Decisional regret is higher in patients opting for MET, which could be attributed to increased anxiety perceived by the patient during the treatment course and a higher need for additional procedures for attaining stone clearance after initially opting for MET. Our study advocates for a deeper engagement with patient experiences to refine shared decision-making processes, which is anticipated to bolster patient satisfaction and minimize decisional regret. The insights gained necessitate further investigation into patient-centric outcomes in the treatment strategies for ureteric calculi. Advancing this research could profoundly influence the evolution of clinical guidelines, ensuring that treatment modalities not only address the physical aspects of ureteric calculi but also consider the psychological impact of the treatment on the wellbeing and quality of life of patients.

## Electronic supplementary material

Below is the link to the electronic supplementary material.

Supplementary Material 1

Supplementary Material 2

## Data Availability

The datasets used and/or analysed during the current study are available from the corresponding author on reasonable request.
