# Supplementary material for: Decision regret analysis in early URSL vs medical expulsive therapy 1 for ureteric calculi ≤ 1cm
Source: World J Urol. 2024 Oct 3;42(1):556. doi: 10.1007/s00345-024-05228-2 (PMC11449965; doi:10.1007/s00345-024-05228-2)
Supplement: Supplementary file 1 — Supplementary Material 1 [file 345_2024_5228_MOESM1_ESM.docx]

Supplementary Figure I : Diagrammatic representation of the study methodology of DRAUMET study
