# Supplementary material for: Decision regret analysis in early URSL vs medical expulsive therapy 1 for ureteric calculi ≤ 1cm
Source: World J Urol. 2024 Oct 3;42(1):556. doi: 10.1007/s00345-024-05228-2 (PMC11449965; doi:10.1007/s00345-024-05228-2)
Supplement: Supplementary file 2 — Supplementary Material 2 [file 345_2024_5228_MOESM2_ESM.docx]

**Supplementary Table II: Propensity score matched analysis based on age and stone size**

| **Parameters** | **Group** | | **p value** |
| --- | --- | --- | --- |
|  | **URSL** | **MET** |  |
| **Number of patients (n)** | 111 | 111 |  |
| **Age (Years)** | 39.07 ± 14.17 | 39.69 ± 14.03 | 0.725^1^ |
| **Stone size (mm)** | 5.78 ± 1.53 | 5.50 ± 1.89 | 0.372^1^ |
| **Stone Clearance at 4 Weeks (Yes)*** | 101 (91.8%) | 76 (68.5%) | <0.001^2^ |
| **Need For Additional Procedure (Yes)*** | 1 (0.9%) | 35 (31.5%) | <0.001^2^ |
| **Decisional Conflict (Yes)*** | 88 (79.3%) | 64 (57.7%) | <0.001^2^ |
| **CUSP Questionnaire** |  |  |  |
| *Pain** | 15.67 ± 3.72 | 16.51 ± 4.20 | 0.013^1^ |
| *Fatigue* | 10.03 ± 2.35 | 10.44 ± 2.97 | 0.359^1^ |
| *Alteration in Work, Daily Activities & Travel Plans** | 6.39 ± 1.53 | 6.05 ± 1.72 | 0.027^1^ |
| *Sleep Disturbance* | 7.71 ± 1.68 | 7.78 ± 2.15 | 0.845^1^ |
| *Anxiety** | 5.68 ± 1.40 | 6.94 ± 1.89 | <0.001^1^ |
| *Urinary Symptoms** | 5.95 ± 1.43 | 5.59 ± 1.46 | 0.048^1^ |
| **Decision Regret Score*** | 15.45 ± 9.45 | 33.24 ± 30.89 | 0.004^1^ |
| **OPTION Tool score** | 72.01 ± 9.45 | 71.99 ± 9.64 | 1.000^1^ |
| **Propensity Score** | 0.30 ± 0.14 | 0.36 ± 0.21 | 0.151^1^ |
| ***Table legends:*** URSL: Ureteroscopy Lithotripsy; MET: Medical Expulsive Therapy; OQ: OPTION Questionnaire; HRQoL: Health-Related Quality of Life; mm: Millimeters; NA: Not Applicable  *1: Wilcoxon-Mann-Whitney U Test, 2: Chi-Squared Test*  * Statistically significant at p < 0.05 | | | |

**Supplementary Table III:**

**Stone size specific sub-group analysis of the outcomes in URSL and MET groups**

| **Parameters** | **Group** | | **p value** |
| --- | --- | --- | --- |
|  | **URSL** | **MET** |  |
| ***Stone size ≤ 5mm*** | | | |
| **Number of patients (n)** | 88 | 55 |  |
| **Age (Years)** | 38.86 ± 14.42 | 37.11 ± 13.34 | 0.518^1^ |
| **Opt-Out Of MET Due To Recurrent Pain** | - | 9 (16.4%) | 1.000^2^ |
| **Stone Cleared at 4 Weeks (Yes)*** | 85 (96.6%) | 42 (76.4%) | <0.001^2^ |
| **Need For Additional Procedure (Yes)*** | 3 (3.4%) | 13 (23.6%) | <0.001^2^ |
| **Decisional Conflict (Yes)*** | 65 (73.9%) | 31 (56.4%) | 0.030^2^ |
| **CUSP Questionnaire** |  |  |  |
| *Pain* | 15.73 ± 3.88 | 15.71 ± 3.88 | 0.447^1^ |
| *Fatigue* | 10.08 ± 2.42 | 9.89 ± 2.62 | 0.707^1^ |
| *Alteration in Work, Daily Activities & Travel Plans** | 6.53 ± 1.57 | 5.76 ± 1.66 | 0.001^1^ |
| *Sleep Disturbance* | 7.82 ± 1.79 | 7.45 ± 1.86 | 0.246^1^ |
| *Anxiety** | 5.74 ± 1.52 | 7.16 ± 1.81 | <0.001^1^ |
| *Urinary Symptoms** | 5.98 ± 1.44 | 5.15 ± 1.10 | <0.001^1^ |
| **Decision Regret Score*** | 15.00 ± 11.67 | 28.18 ± 28.74 | 0.001^1^ |
| **OPTION Tool score** | 72.45 ± 9.93 | 70.67 ± 9.32 | 0.133^1^ |
| ***Stone size >5mm*** | | | |
| **Number of patients (n)** | 308 | 56 |  |
| **Age (Years)** | 38.54 ± 13.31 | 42.23 ± 14.35 | 0.083^1^ |
| **Opt-Out Of MTE Due To Recurrent Pain** | - | 7 (12.5%) | - |
| **Stone Cleared at 4 Weeks (Yes)** | 258 (84.3%) | 34 (60.7%) | <0.001^2^ |
| **Need For Additional Procedure (Yes)** | 9 (2.9%) | 22 (39.3%) | <0.001^3^ |
| **Decisional Conflict (Yes)** | 241 (78.2%) | 33 (58.9%) | 0.002^2^ |
| **CUSP Questionnaire** |  |  |  |
| *Pain** | 16.30 ± 4.06 | 17.30 ± 4.39 | 0.005^1^ |
| *Fatigue* | 10.46 ± 2.49 | 10.98 ± 3.21 | 0.403^1^ |
| *Alteration in Work, Daily Activities & Travel Plans* | 6.56 ± 1.60 | 6.32 ± 1.75 | 0.157^1^ |
| *Sleep Disturbance* | 8.02 ± 1.97 | 8.11 ± 2.37 | 0.961^1^ |
| *Anxiety** | 5.88 ± 1.54 | 6.71 ± 1.95 | 0.001^1^ |
| *Urinary Symptoms* | 6.28 ± 1.60 | 6.04 ± 1.64 | 0.345^1^ |
| **Decision Regret Score*** | 17.91 ± 13.20 | 38.21 ± 32.35 | 0.001^1^ |
| **OPTION Tool score** | 71.76 ± 9.47 | 73.29 ± 9.85 | 0.127^1^ |
| ***Table legends:*** URSL: Ureteroscopy Lithotripsy; MET: Medical Expulsive Therapy; OQ: OPTION Questionnaire; HRQoL: Health-Related Quality of Life; mm: Millimeters; NA: Not Applicable  *1: Wilcoxon-Mann-Whitney U Test, 2: Chi-Squared Test*  * Statistically significant at p < 0.05 | | | |
